# Anti-inflammatory, antioxidant, and antimicrobial activities of *Cocos nucifera* var. *typica*

**DOI:** 10.1186/1472-6882-13-107

**Published:** 2013-05-16

**Authors:** Rafaela Ribeiro Silva, Davi Oliveira e Silva, Humberto Rollemberg Fontes, Celuta Sales Alviano, Patricia Dias Fernandes, Daniela Sales Alviano

**Affiliations:** 1 Universidade Federal do Rio de Janeiro, Instituto de Microbiologia Paulo de Góes, Laboratório de Estruturas de Superfície de Micro-organismos, Instituto de Microbiologia Paulo de Góes, Rio de Janiero, Brasil; 2 Universidade Federal do Rio de Janeiro, Instituto de Ciências Biomédicas, Laboratório de Farmacologia da Dor e da Inflamação, Rio de Janeiro, Brasil; 3 Embrapa Tabuleiros Costeiros, Av. Beira Mar 3250 Caixa postal 44. Cep 49015-040, Bairro Treze de Julho, Aracaju, Sergipe, Brasil

**Keywords:** *Cocos nucifera*, Anti-inflammatory activity, Antioxidant activity, Antimicrobial activity

## Abstract

**Background:**

Teas from the husk fiber of *Cocos nucifera* are used in the folk medicine to treat arthritis and other inflammatory processes. Some works show that some varieties have biological activities. However, one of the main variety of the species, *C*. *nucifera* var. *typica*, known in Brazil as “gigante”, was not studied yet. Thus, this study evaluates if this variety has the anti-inflammatory and antimicrobial activities already reported in other varieties.

**Methods:**

*C*. *nucifera* aqueous crude extract (10, 50, and 100 mg/kg) and the reference drugs morphine (1 mg/kg) and acetylsalicylic acid (100 mg/kg) were evaluated in models of inflammation (formalin-induced licking and subcutaneous air pouch). The antioxidant activity was evaluated by 2,2-diphenyl-1-picryl-hydrazyl-hydrate (DPPH) photometric assay and compared with those of the standards (quercetin, rutin, and ascorbic acid). The extract was also screened against *Candida albicans*, *Escherichia coli*, *Staphylococcus aureus,* and methicillin-resistant *Staphylococcus aureus* (MRSA), in the agar diffusion method. The minimal inhibitory concentration (MIC) and minimal bactericidal concentration (MBC) were determined by the broth micro-dilution assay. Activities of combinations of the extract and antibiotics (methicillin or vancomycin) against MRSA were evaluated using checkerboard assays.

**Results:**

The extract significantly inhibited the time that the animals spent licking the formalin-injected paws (second phase). The extract also inhibited the inflammatory process induced by subcutaneous carrageenan injection by reducing cell migration, protein extravasation, and TNF-α production. Additionally, the extract showed an antioxidant potential *in vitro* as good as standards in their antioxidant activity. The extract was active only against *S*. *aureus* and MRSA. MIC and the bactericidal concentrations were identical (1,024 μg/ml). The extract and methicillin acted synergistically against the clinical MRSA isolate, whereas an indifferent effect was detected when the extract was combined with vancomycin.

**Conclusions:**

The extract exhibits anti-inflammatory activity through the inhibition of the cell migration. The mixture of extract constituents and methicillin could lead to the development of a new combination antibiotic against MRSA infections.

## Background

The varieties of *Cocos nucifera* are found all over the tropical regions. In Brazil, there are different varieties like the “gigante” [1]. The aqueous crude extract of husk fiber from *C*. *nucifera* is widely used in northeastern Brazilian traditional medicine to treat diarrhea and arthritis [2]. During the past years, it has been shown that the “comum” *C*. *nucifera* has antibacterial, antiviral [2], antioxidant [3], anti-neoplastic [4,5], and anti-inflammatory activities [6].

The variety “anão” is divided in “green”, “yellow” and “red”. The “mestiço” are known as segregated hybrids, resulting from aleatory cross between diverse plants, and thereby with unknown origin [1].

The inflammatory process is a complex phenomenon from vascular tissues to harmful stimuli, such as pathogens, damaged cells or irritants [7]. In this paper, the anti-inflammatory action of an aqueous crude extract of husk fiber from *C*. *nucifera* was assessed in two animal models. Since, reactive oxygen species (ROS) have been implicated as novel second messengers that help regulate cellular events like inflammation [8], the free radical scavenging properties was either evaluated.

Besides the antioxidant and anti-inflammatory activities, the antimicrobial action was also evaluated by antibacterial and antifungal activities assays in this study, once the antimicrobial properties of the aqueous extracts of husk fiber from another Brazilian and Nigerian *C*. *nucifera* varieties were previously described [2,9]. Since combination therapies may results in the administration of a lower dose of commercial antimicrobials, which might reduce drug toxicity and improve efficacy [10], combinations of the *C*. *nucifera* extract and commercial antimicrobials were also performed in this paper.

Considering that previous studies demonstrated the low toxicity of aqueous crude extract of husk fiber from other varieties of *C*. *nucifera*[3,6], this is also expected for the “gigante” variety. In the popular use of this plant extract, the occurrence of adverse effects is not documented and aqueous crude extracts generally have low acute toxicities [11].

Furthermore, the use of the husk fiber might lead to the production of novel low-cost therapies and adjunct treatments because it is a by-product from the processing of *C*. *nucifera*[6]. Therefore, the purpose of this study was to evaluate the anti-inflammatory, antioxidant and antibacterial activities of a variety of *C*. *nucifera* that has not been investigated yet, the “gigante”.

## Materials and methods

### Plant material

*C*. *nucifera* (Arecaceae) var. *typica*, commonly known as “gigante”, was collected in Sergipe, Brazil (Campo Experimental de Itaporanga, Embrapa Tabuleiros Costeiros) and authenticated by the forest engineer Humberto Rollemberg Fontes (Fitotecnia researcher). The characterization and identification of this variety was done following morphological, phenological, and production aspects of the plant, in addition to other criteria, such as age of the plant [12]. A voucher specimen was deposited (number ASE- 27,441) at the Federal University of Sergipe.

### Preparation of *C. Nucifera* crude extract

The water extract from husk fiber was prepared by infusion, as described previously [2]. The use of aqueous extract was in accordance with the popular medicinal knowledge. The extract was filtered, lyophilized and stored at −20^o^C, yielding around 10% of the dry weight of starting material. For the experiments described below, the aqueous crude extract was re-suspended in distilled water. In the case of the antimicrobial assays, it was sterilized by filtration using a 0.22 μm membrane.

### Phytochemical composition

The lyophilized crude aqueous extract was submitted to HPLC/DAD analyses, according to a protocol devised by Peng et al. [13] to analyze procyanidins in grape seeds. Samples (20 μl) were loaded onto a 4.6 x 250 mm reverse phase RP-18 column (250 mm X 4,6 mm X 5 μm; XTerra®). Elution was achieved with solvents A (0.02% phosphoric acid in water) and B (82% acetonitrile, 0.04% phosphoric acid) as follows: 0 to 15% solvent B in the first 15 min, 15 to 16% from 15 to 40 min, 16 to 17% from 40 to 45 min, 17 to 43% from 45 to 48 min, 43 to 52% from 48 to 49 min, held isocratic at 52% from 49 to 56 min, reduced from 52 to 43% from 56 to 57 min, and from 17 to 0% from 58 to 60 min. Peaks were detected at 280 nm.

The concentration of procyanidins in the sample solution of *C*. *nucifera* lyophilized crude aqueous extract was determined by the vanillin-HCl assay [14]. Briefly, 2.5 ml of methanol (control) or 1% vanillin solution in methanol (sample) and 2.5 ml of 9 M HCl in methanol was added to a test tube containing 1 ml of catechin solution (0 to 300 μg/ml in methanol) or test solution (150 to 250 μg/ml polyphenols in methanol). The reaction mixture was incubated for 20 min at 30°C, and the absorbance at 500 nm was measured. The absorbance was calculated as follows for each standard and sample solution: a calibration curve was prepared using the calculated absorbance for the catechin solution, and the total procyanidin in each test solution was calculated from the calibration curve.

### Animals

All experiments were performed with male Swiss mice (20–25 g) obtained from our own animal facility. Animals were maintained in temperature-controlled room (22±2°C) with a 12 h light/dark cycles and free access to food and water. Twelve hours prior to each experiment, the animals received only water in order to avoid food interfering with the substance absorption. Animal care and research protocols (ICBDFBC-015) were in accordance with the principles and guidelines adopted by the Brazilian College of Animal Experimentation (COBEA), approved by the Ethical Committee for Animal Research (Biomedical Science Institute/UFRJ).

### Drugs and extract administration

Acetylsalicylic acid (ASA) and morphine hydrochloride were purchased from Sigma (St. Louis, MO, USA) and Merck Inc. (Brazil), respectively. They were dissolved in sterile water just before use. The extract was dissolved in sterile water and administered by oral gavage at doses of 10, 50, and 100 mg/kg in a final volume of 0.1 ml. Morphine (1 mg/kg) and ASA (100 mg/kg) were used as reference drugs and were also administered by oral gavage. The negative control group received vehicle by oral gavage.

### Formalin test

The formalin test was conducted in a manner similar to that described by Gomes et al. [15]. Animals received an injection of 20 μl of formalin (2.5% v/v) into the dorsal surface of the left hind paw. The time that the animal spent licking the injected paw was recorded. The response consists of two phases. The first phase occurs 5 min immediately following the formalin injection (neurogenic pain response), and the second phase occurs 15–30 min after formalin injection (inflammatory pain response). The animals were pretreated with oral doses of extract, ASA, morphine or vehicle 60 min before the injection of formalin.

### Subcutaneous air pouch (SAP) model

The method was similar to that described by Romano et al. [16] with several modifications described in Raymundo et al. [17]. Briefly, air pouches were produced by subcutaneous injection of sterile air (10 ml) into the intrascapular region of the mice. After three days, another injection of air (10 ml) was performed in order to maintain the pouches. Three days after the last injection of air, animals received an injection of sterile carrageenan suspension (1%) into the SAP. Mice were pre-treated with oral doses of extract, ASA or vehicle 1 h before and 23 h after carrageenan injection in the SAP. Animals were sacrificed 24 h after carrageenan injection and the cavity was washed with 1 ml of sterile PBS. The total number of cells was determined with the aid of a haemocytometer. The exudates were centrifuged at 170 x g for 10 min at 4°C, and the supernatants were collected and stored at −20°C to further analysis. Supernatants from exudates collected in the SAP were used to measure tumor necrosis factor-α (TNF-α) and protein. TNF-α was quantified by enzyme-linked immunosorbent assay (ELISA), using the protocol supplied by the manufacturer (B&D, USA). The protein content of each supernatant was determined using the BCA method (BCA^TM^ Protein Assay Kit, Pierce).

### Antioxidant activity determined by the 2,2-diphenyl-1-picryl-hydrazyl-hydrate (DPPH) photometric assay

The free radical scavenging activity of the *C*. *nucifera* extract was evaluated as described by Mensor et al. [18]. Briefly, the plant extract was mixed with a 0.3 mM 2,2-Diphenyl-1-picryl-hydrazyl-hydrate (DPPH) ethanol solution, to give final concentrations of 0.78, 1.56, 3.13, 6.25, 12.5, 25, 50, and 100 μg of extract per ml of DPPH solution. After 30 min at room temperature, the absorbance values were measured at 518 nm and converted into the percentage of antioxidant activity. Free radical scavenging activity of the extract was compared with those of the quercetin, rutin, and ascorbic acid.

### Microorganisms and antimicrobial standards

Methicillin and vancomicin were obtained from Sigma-Aldrich (Brazil) and stored according to the supplier’s instructions. The following reference strains were used: *Staphylococcus aureus* ATCC 6538 and the fungus *Candida albicans* ATCC 36802. The study also included a methicillin-resistant *Staphylococcus aureus* strain (BMB 9393) and a clinical isolate of *Escherichia coli* obtained from University Hospital of the Federal University of Rio de Janeiro.

### Antimicrobial activities

The antimicrobial activity of the *C*. *nucifera* extract was evaluated by the agar diffusion method described by Hili et al. [19]. Microorganisms (2 x 10^5^ cells) were spread over appropriate plate (Brain Heart Infusion agar for bacteria and Sabouraud agar for the fungus). The extract was diluted in water (10 mg/ml), sterilized by filtration, and 10 μl aliquots of aqueous crude extract were applied to newly inoculated plates. Plates were incubated at 37°C for 24 h (bacteria) or for 48 h at room temperature (fungus).

### Minimal inhibitory concentration (MIC) and minimal bactericidal concentration (MBC) assays

The MICs values of the extract against the test microorganisms were determined by broth microdilution method as recommended by CLSi [20]. The microdilution method was also used to determine MBC values. The substances were transferred to a microplate in order to obtain two-fold serial dilutions of the original substance. Then, an inoculum (10 μl) containing 5×10^6^ CFU/ml was added to each well and the microplates were aerobically incubated at 37°C for 24 h. Wells without inoculum added were used for sterility control, and the positive controls comprised of inoculated growth medium without the substances.

Bacterial growth was indicated by the presence of turbidity and a pellet on the well bottom, which was confirmed with 30 μl of resazurin (Sigma-Aldrich) added aseptically and incubated at 37°C for 1 h. The MIC was defined as the lowest extract concentration that was able to completely inhibit the bacterial growth. Methicillin and vancomicin were used as antimicrobial standards.

To evaluate whether the action of the extract was microbiostatic or microbicidal, 20 μl of the microbial culture were removed from wells with concentrations equal to, or higher than, the MIC, inoculated on BHI agar plates, and incubated at 37°C for 24 h. The MBC was defined as the lowest extract concentration at which all of the bacteria have been killed.

### Synergistic activity of the extract and antimicrobial drugs against microorganisms

According to Mahboubi and Bidgoli [21], synergistic antimicrobial activity was evaluated by a checkerboard assay with the broth microdilution method. The extract and commercial antimicrobials were combined in concentrations lower than their individual MIC values by serial dilution in microplates. An inoculum (10 μl) containing 5×10^6^ CFU/ml was added to each well and the microplates were incubated at 37°C for 24 h. The visual bacterial growth was confirmed with 30 μl of resazurin (Sigma-Aldrich) added aseptically and incubated at 37°C for 1 h.

Fractional inhibitory concentrations (FICs) were calculated as the MIC of the combination of the extract and a commercial antimicrobial divided by the MIC achieved when one of them was used individually. FIC *index* is widely accepted for use in evaluating *in vitro* synergistic actions. It is the sum of FICs values and indicates the nature of an interaction between two compounds. A FIC *index* between 0.5 and 4.0 indicates an insignificant interaction, whereas FIC *index* values <0.5 and >4.0 have synergistic and antagonistic interactions, respectively.

### Statistical analysis

*In vitro* experiments (antimicrobial and antioxidant assays) were undertaken in triplicate sets. All animal groups were composed of 6–10 mice and the results are presented as the mean ± S.D. Statistical significance between groups was determined by analyses of variance (ANOVA) followed by Bonferroni’s test. *p* values less than 0.05 (*p< 0.05) were considered significant.

## Results and discussion

### Phytochemical composition

**Figure 1:**
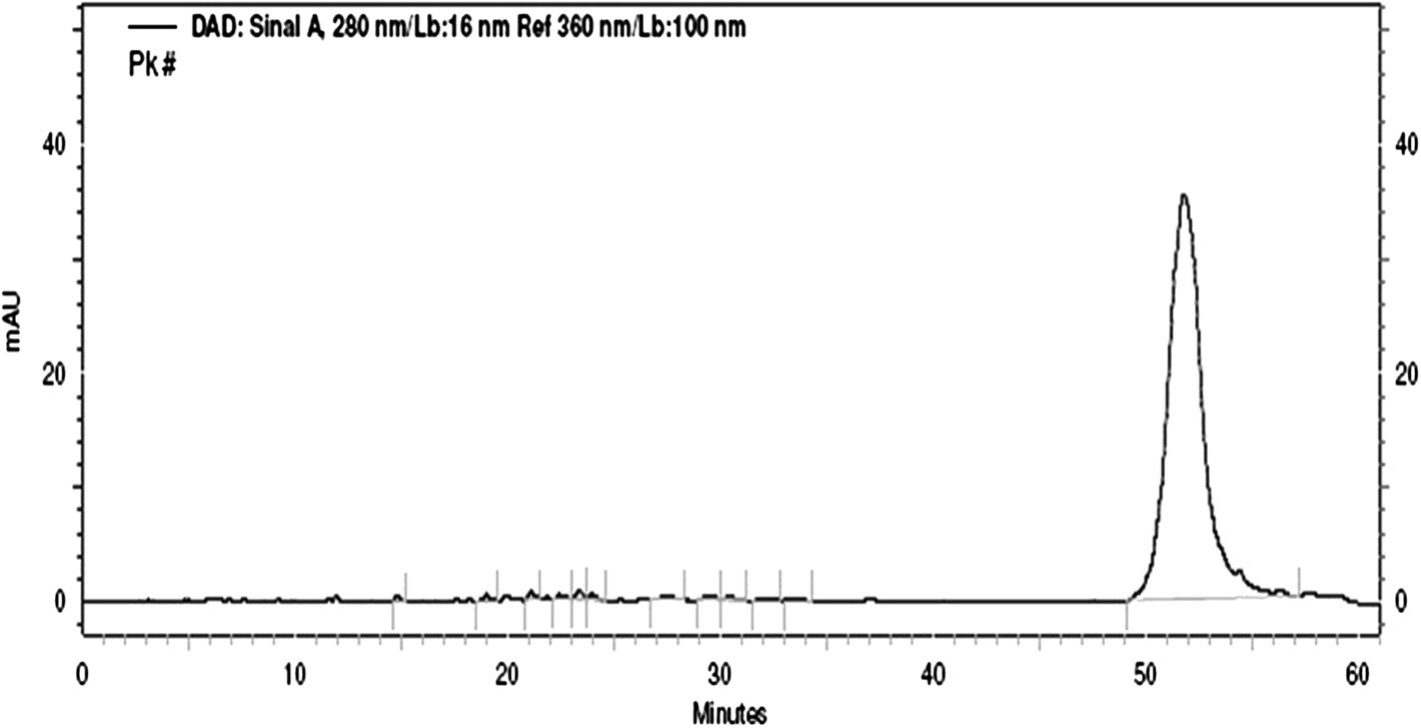
**A trace of reverse phase HPLC analysis of *C. nucifera *lyophilized crude aqueous extract.** The asterisk (*) in the figure indicates polymeric procyanidins.

Application of the reverse phase C18 HPLC protocol devised by Peng et al. [13] to the crude aqueous extract produced the chromatogram shown in Figure 1. The broad peak at 50–55 min belongs to a mixture of polymeric procyanidins. This elution behavior and UV absorption characteristics indicate the presence of condensed tannins [13].

In order to corroborate the presence of these tannins, the vanillin-HCl assay was performed. This test is highly specific for procyanidins and involves acidic hydrolysis of the condensed tannins followed by coupling of the flavanol units to vanillin. Polymeric procyanidins were detected at a concentration of 103.80 ± 10.5 mg/g of aqueous extract.

### Anti-inflammatory activity

Initially, the anti-inflammatory effect of the extract was evaluated by the formalin-induced licking model. The response consists of two phases and can evaluate both antinociceptive and anti-inflammatory activities. Centrally acting drugs, such as narcotics, are able to inhibit the two phases equally. The first phase involves direct chemical stimulation of nociceptive afferent fibers, leading to acute neurogenic pain. However, the second phase involves inflammation in the paw and central sensitization [22,23].

**Figure 2:**
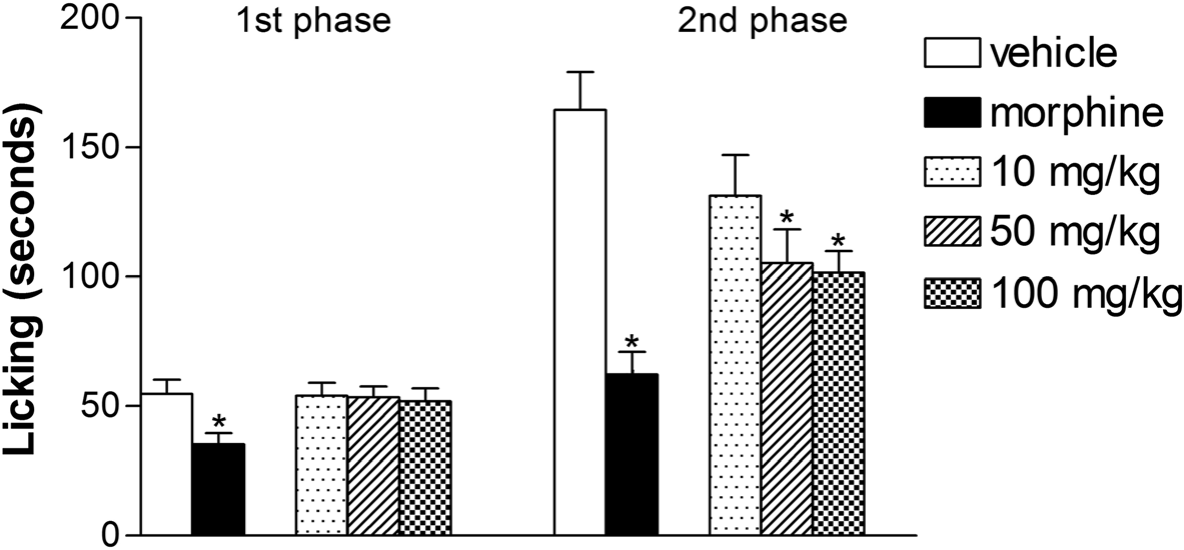
**Effects of the *C. nucifera *extract on formalin**-**induced licking in mice.** Animals were pretreated by oral administration different doses of the *C*. *nucifera* extract, morphine (1 mg/kg), or vehicle. The results are presented as mean ± S.D. (n = 6–10) of the time that the animals spent licking the formalin-injected paws. Statistical significance was calculated by ANOVA followed by Bonferroni’s test. * indicates p < 0.05 when comparing to the vehicle-treated group.

As depicted in Figure 2, extract treatment did not reduce the time that the animal spent licking the formalin-injected paw during the first phase (neurogenic pain response). However, during the second phase (inflammatory pain response), extract treatment induced dose-dependent inhibition, indicating an anti-inflammatory effect. Therefore, the extract could be acting through inhibition of the formation and/or liberation of inflammatory mediators or by directly blocking its receptor. In order to evaluate how the extract could inhibit the inflammatory reaction, another model of inflammation was performed, the subcutaneous air pouch. This model was not achieved by previous studies with aqueous extract of husk fiber from *C*. *nucifera*. In this model, subcutaneous injections of air into the back result in formation of a cavity similar to the synovium and the injection of carrageenan in this cavity induces inflammation. The site serves as a reservoir of cells and mediators that can be measured in the locally accumulating fluid [24].

**Figure 3:**
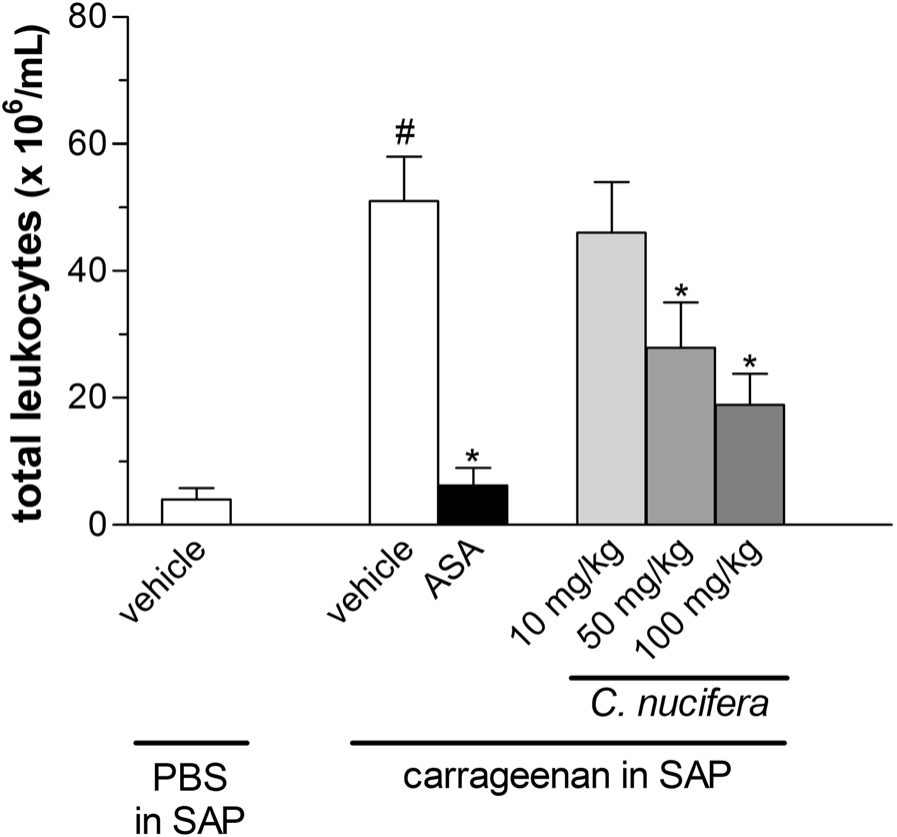
**Effect of the *C. nucifera *extract in the subcutaneous air pouch (SAP) model.** Animals were pretreated by oral administration with different doses of the extract 24 h and 1 h prior to carrageenan (1%) injection into the SAP. The results are presented as mean ± S.D. (n = 6–10) of total leukocytes (×10^6^/ml). Statistical significance was calculated by ANOVA followed by Bonferroni’s test. * indicates p < 0.05 when comparing *C*. *nucifera*-treated mice with the vehicle-treated group; # indicates p < 0.05 when comparing vehicle-treated mice to the PBS-treated group.

Injection of carrageenan (1%) into the mice air pouches increased the number of leukocytes that migrated to the cavity when compared to the control that received PBS in the SAP. Pre-treatment with lower dose of the extract (10 mg/kg) was not able to significantly reduce the number of leukocytes. However, pre-treatment with the other doses of extract (50 or 100 mg/kg) significantly suppressed the cell migration (Figure 3).

**Figure 4:**
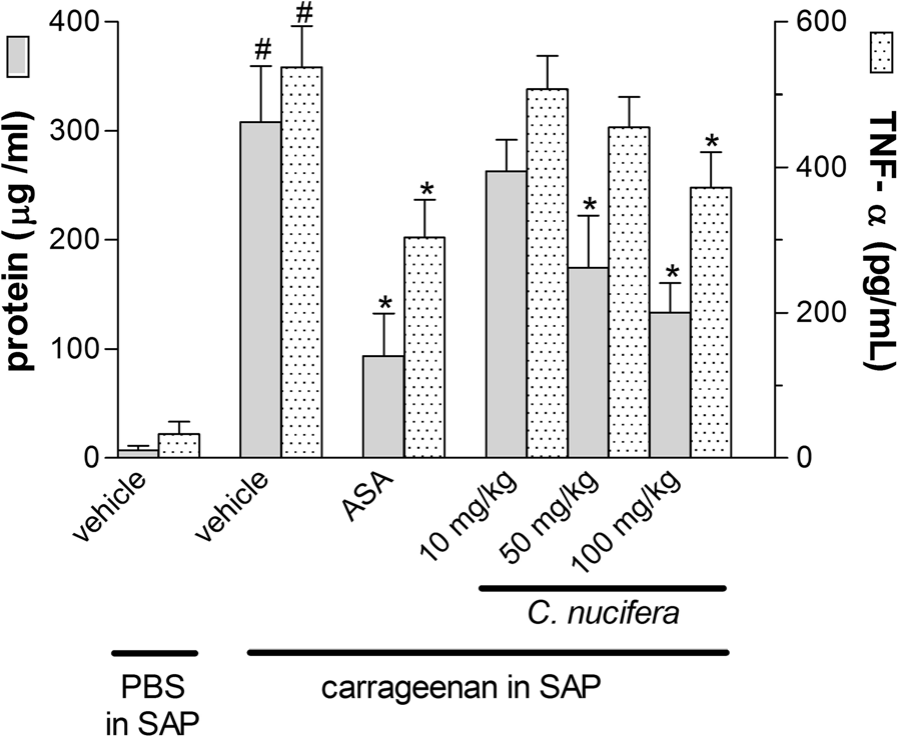
**Effect of the *C. nucifera *extract on protein leakage and TNF**-**α production.** Animals were pretreated by oral administration with different doses of the extract 24 h and 1 h prior to carrageenan (1%) injection into the subcutaneous air pouch (SAP). The results are presented as mean ± S.D. (n = 6–10) of protein (μg/ml) or TNF-α (pg/ml). Statistical significance was calculated by ANOVA followed by Bonferroni’s test. * indicates p < 0.05 when comparing *C*. *nucifera*-treated mice with the vehicle treated group; # indicates p < 0.05 when comparing vehicle-treated mice with the PBS-treated group.

A similar pattern was observed in the exudates protein concentrations. An increase in the levels of protein was detected following carrageenan injection in the SAP. This carrageenan-induced protein leakage was significantly inhibited by pre-treatment with the higher doses of extract (50 or 100 mg/kg) (Figure 4). These effects could be due to disruption of inflammatory mediators’ formation or by inhibition of receptor function.

One of the mediators that play an important role in the inflammatory process is TNF-α. It is released from activated monocytes and macrophages and increases vascular endothelial permeability [25]. The production of this mediator was dramatically increased by the injection of carrageenan in the SAP. Nevertheless, the levels of this mediator in the exudates were significantly reduced in mice that received pre-treatment with the highest dose of extract (100 mg/kg) (Figure 4).

### In vitro antioxidant activity

**Figure 5:**
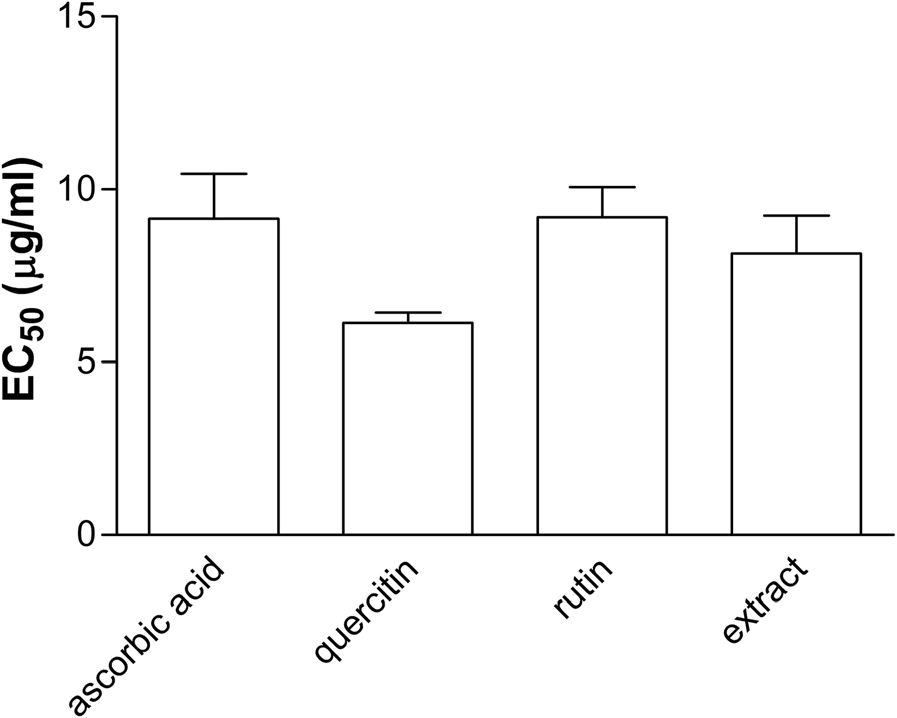
**Free radical scavenging properties of the *C. nucifera *extract, using the DPPH photometric assay.** The results are presented as mean ± S.D. from three independent assays. EC50 values were determined.

The antioxidant activity was determined by the 2,2-diphenyl-1-picryl-hydrazyl-hydrate (DPPH) assay. The EC_50_ values were calculated, and the result obtained for the *C*. *nucifera* extract was comparable with those of the standards (quercetin, rutin, and ascorbic acid) (Figure 5).

A good antioxidant effect of the aqueous crude extract of husk fiber from “olho de cravo” *C*. *nucifera* has already been determined by the same model (EC_50_= 10.0 ± 0.7 μg/ml). This activity could be attributed to the presence of condensed tannins in the husk fiber from *C*. *nucifera*[11].

Nevertheless, the extrapolation of an antioxidant effect *in vitro* to an *in vivo* action is not trivial. A direct antioxidant activity of flavonoids *in vivo* is likely to be limited to tissues in which relatively high concentrations of these compounds could be achieved, like in the digestive tract. In lower concentrations, they could support indirect antioxidant actions, like regulation of pro-oxidant enzymes and inhibition of protein–receptor coupling that initiates oxidant production [26].

However, the antioxidant activity can be related to an anti-inflammatory effect. Free radicals can attract inflammatory mediators contributing to a generalized inflammatory response [27]. The reactive oxygen species are linked to increased endothelial permeability and leukocyte transendothelial migration [28]. This can explain the inhibition of the carrageenan-induced protein leakage and the leukocyte migration by the extract of *C*. *nucifera* in the SAP.

### Antimicrobial activity

The results of the agar diffusion assay showed that the extract does not have an antimicrobial effect against the fungus and the Gram-negative bacterium tested. However, an inhibition zone was observed when the extract was tested against *S*. *aureus* and MRSA (Table 1).

**Table 1:** **Antimicrobial screening of *C***. ***nucifera *extract by agar diffusion method**

**Microorganism**	**Antimicrobial screening**
*C*. *albicans*	-
*E*. *coli*	-
*S*. *aureus*	+
MRSA	+

(-) no activity; (+) activity.

Esquenazi et al. [2] found that the “olho de cravo” *C*. *nucifera* has activity against one *S*. *aureus* ATCC strain and three clinical isolates of *S*. *aureus*. Further, Akinyele et al. [9] studied the aqueous extracts of the husk fiber from a *C*. *nucifera* variety from Nigeria. They demonstrated that it also has activity against *S*. *aureus*, in addition to inhibiting Gram-negative bacteria, such as *Escherichia coli*.

In accordance to the lack of reported activity of *C*. *nucifera* against *Candida albicans* in the literature, the “gigante” variety does not have effect against this fungus. Despite of being the same species of coconut, the different varieties have different types and quantities of substances in the fiber. The biological activity can be affected by structural variations, such as stereochemistry [29].

This may be the reason why the “gigante” variety does not have the same effects of the variety from Nigeria described by Akinyele et al. [9]. In the present paper, the extract does not possess growth inhibitory activity against *Escherichia coli*. Nevertheless, it is important to note that the tested strain is different from that of the other study. Discrepancy in findings may be the result of the pathogen strains utilized or also be due to the variety of the plant and extraction process used.

Others varieties have activity against *S*. *aureus* and the “gigante” variety is likewise active. It was also investigated if it would be able to inhibit the MRSA growth and it was. Thus, the minimal inhibitory concentrations of the extract against those bacteria (*S*. *aureus* and MRSA) were determined and results are shown in Table 2.

**Table 2:** **Minimal inhibitory concentrations** (**MICs**) **of *C***. ***nucifera *extract and antimicrobial drugs by the micro**-**dilution method**

**Microorganism**	MIC* (μg/mL)		
	**extract**	**MET**	**VCM**
*S*. *aureus*	1024	0,25	nd
MRSA	1024	512	1

* All MICs were microbicidal; MET - methicillin; VCM - vancomycin; nd - not determined.

MIC was the same for the two bacteria strains tested (1024 μg/ml) and at this concentration, a microbicidal effect was observed. MIC values were also determined for methicillin. Methicillin-resistant staphylococcal is defined as MIC ≥ 4 μg/ml [30]. To the methicillin-resistant strain, vancomicin was used as standard antimicrobial.

Despite having been able to killing the two strains of bacteria, the concentration of the extract was higher than those of the standards antimicrobial used. However, the MIC must be interpreted in the appropriate context. The raw extract is a mixture of active and non-active compounds and therefore higher MICs are expected [31].

Cos et al. [32] proposed an endpoint with concentration required to produce 50% growth inhibition below 100 μg/ml for extracts. Nevertheless, in the present study it was evaluated the extract’s concentration that was able to completely inhibit the bacterial growth and not only 50% growth inhibition. Thus, a higher MIC is acceptable. Webster et al. [31] interpreted a MIC value of crude plant extracts approximately to 1000 μg/ml as showing strong antifungal potential.

The antimicrobial activity may be enhanced by synergistic effect of natural products and antimicrobial drugs. Therefore, the checkerboard assay was performed to evaluate the antimicrobial effects of combinations of antimicrobial drugs and the extract against MRSA. When the extract was combined with vancomycin, the MIC of the extract against MRSA reduced 4-fold, however the MIC of vancomycin against MRSA was almost the same. In this combination, the FIC index was 1.19, corresponding to an indifferent effect (Table 3).

**Table 3:** **Effects of combinations of antimicrobial drugs and *C***. ***nucifera *extract against MRSA**

	**MIC of drug in combination**	**MIC of extract in combination**	**FIC of drug**	**FIC of extract**	**FIC index**
VCM	0,9375	256	0,9375	0,25	1,19 (I)
MET	96	256	0,1875	0,25	0,44 (S)

VCM - vancomicin; MET - methicillin; MIC - minimal inhibitory concentration (μg/ml); FIC -fractional inhibitory concentration; I- indifferent; S – synergistic.

Nevertheless, the MIC value of methicillin against MRSA was lowered from 512 to 96 μg/ml when the extract was added at a concentration of 256 μg/ml (4-fold reduction in MIC). This combination had a FIC index calculated as 0.44, corresponding to a synergistic effect (Table 3). However, this synergistic effect was not able to reverse the methicillin resistance (MIC remained > 4 μg/ml).

The extract can be fractionated in order to check if the activity improved. In addition, the active substances in the extract and their modes of action should be further investigated. Moreover, the extract can lead to the development of novel inexpensive phytomedicines or models for synthetic substances.

The antistaphylococcal effect of the extract is important because MRSA and vancomycin-intermediate *S*. *aureus* are spreading worldwide and the pharmaceutical arsenal available to control antimicrobial-resistant bacteria is limited. Invasive infections by *S*. *aureus* are associated with significant morbidity and mortality, creating urgency in the development of new therapies and adjunct treatments that act against the resistant strains [33,34].

## Conclusions

Concerning the crude nature of the extract, the results allow concluding that it exhibited significant bioactivity and properties that support its uses in the folk medicine. The extract exhibits anti-inflammatory activity through the inhibition of the cell migration. In addition, the mixture of extract constituents and methicillin could lead to the development of a new combination antibiotic against MRSA infections.

Although the modes of action remain to be completely elucidated, the extract and its constituents might be promising candidates for the direct or adjunct treatment of inflammation and infection by MRSA, with low cost.

## Competing interests

The authors declare that they have no competing interests.

## Authors’ contributions

RRS carried out all the experiments and wrote the manuscript; DOS prepared and characterized the *C*. *nucifera* extracts; HRF identificated the *C*. *nucifera* variety, collected the seeds and extracted the fibers; CSA participated in the design of the study and revised it critically for important intellectual content; PDF participated in the design of the pharmacological study, helped to draft the manuscript and revised it; DAS participated in the design and coordination of the microbiological study. All authors read and approved the final manuscript.

## Pre-publication history

The pre-publication history for this paper can be accessed here:

http://www.biomedcentral.com/1472-6882/13/107/prepub

## References

[B1] Adebayo J Santana A Krettli A Evaluation of the antiplasmodial and cytotoxicity potentials of husk fiber extracts from *Cocos nucifera*, a medicinal plant used in Nigeria to treat human malaria Hum ExpToxicol 2012 31 244 249 10.1177/0960327111424298 22241625

[B2] Esquenazi D Wigg MD Miranda MM Rodrigues HM Tostes JB Rozental S Da Silva AJ Alviano CS Antimicrobial and antiviral activities of polyphenolics from Cocos nucifera Linn. (Palmae) husk fiber extract Res Microbiol 2002 153 647 652 10.1016/S0923-2508(02)01377-3 12558183

[B3] Alviano DS Rodrigues KF Leitão SG Rodrigues ML Matheus ME Fernandes PD Antoniolli AR Alviano CS Antinociceptive and free radical scavenging activities of *Cocos nucifera* L. (Palmae) husk fiber aqueous extract J Ethnopharmacol 2004 92 269 273 10.1016/j.jep.2004.03.013 15138011

[B4] Kirszberg C Esquenazi D Alviano CS Rumjanek VM The effect of a catechin-rich extract of *Cocos nucifera* on lymphocytes proliferation Phytother Res 2003 17 1054 1058 10.1002/ptr.1297 14595586

[B5] Koschek PR Alviano DS Alviano CS Gattass CR The husk fiber of *Cocos nucifera* L. (Palmae) is a source of anti-neoplastic activity Braz J Med Biol Res 2007 40 1339 1343 10.1590/S0100-879X2006005000153 17713650

[B6] Rinaldi S Silva DO Bello F Alviano CS Alviano DS Matheus ME Fernandes PD Characterization of the antinociceptive and anti-inflammatory activities from *Cocos nucifera* L. (Palmae) J Ethnopharmacol 2009 122 541 546 10.1016/j.jep.2009.01.024 19429325

[B7] Ferrero-Miliani L Nielsen OH Andersen PS Girardin SE Chronic inflammation: importance of NOD2 and NALP3 in interleukin-1beta generation Clin ExpImmunol 2007 147 227 235 10.1111/j.1365-2249.2006.03261.x PMC1810472 17223962

[B8] Hordijk PL Endothelial signaling events during leukocyte transmigration FEBS J 2006 273 4408 4415 10.1111/j.1742-4658.2006.05440.x 16956370

[B9] Akinyele TA Okoh OO Akinpelu DA Okoh AI *In vitro* antibacterial properties of crude aqueous and n-hexane extracts of the husk of *Cocos nucifera* Molecules 2011 16 2135 2145 10.3390/molecules16032135 21372760 PMC6259629

[B10] Lin RD Chin YP Hou WC Lee MH The effects of antibiotics combined with natural polyphenols against clinical methicillin-resistant *Staphylococcus aureus* (MRSA) PlantaMedica 2008 74 840 846 10.1055/s-2008-1074559 18546080

[B11] Alviano WS Alviano DS Diniz CG Antoniolli AR Alviano CS Farias LM Carvalho MA Souza MM Bolognese AM *In vitro* antioxidant potential of medicinal plant extracts and their activities against oral bacteria based on Brazilian folk medicine Arch Oral Biol 2008 53 545 552 10.1016/j.archoralbio.2007.12.001 18243157

[B12] Aragão WM Tupinambá EA Angelo PCS Ribeiro FE Queiroz MA, Goedert CO, Ramos SSR Seleção de cultivares de coqueiro para diferentes ecossistemas do Brasil Recursos Genéticos e Melhoramento de Plantas para o Nordeste Brasileiro (online) 1999 Petrolina: Embrapa Semi-Árido/Embrapa Recursos Genéticos e Biotecnologia

[B13] Peng Z Hayasaka Y Iland PG Sefton M Høj P Waters EJ Quantitative analysis of polymeric procyanidins (Tannins) from grape (*Vitis vinifera*) seeds by reverse phase high-performance liquid chromatography J Agric Food Chem 2001 49 26 31 10.1021/jf000670o 11170555

[B14] Nakamura Y Tsuji S Tonogai Y Analysis of Proanthocyanidins in Grape Seed Extracts, Health Foods and Grape Seed Oils J Heal Sci 2003 49 45 54 10.1248/jhs.49.45

[B15] Gomes NM Rezende CM Fontes SP Matheus ME Pinto AC Fernandes PD Characterization of the antinociceptive and anti-inflammatory activities of fractions obtained from *Copaifera multijuga* Hayne J Ethnopharmacol 2010 128 177 183 10.1016/j.jep.2010.01.005 20064592

[B16] Romano M Faggioni R Sironi M Sacco S Echtenacher B Di Santo E Salmona M Ghezzi P Carrageenan-induced acute inflammation in the mouse air pouch synovial model. Role of tumour necrosis factor Med Inflam 1997 6 32 38 10.1080/09629359791901 PMC2365839 18472831

[B17] Raymundo LJ Guilhon CC Alviano DS Matheus ME Antoniolli AR Cavalcanti SC Alves PB Alviano CS Fernandes PD Characterization of the anti-inflammatory and antinociceptive activities of the *Hyptis pectinata* (L.) Poit essential oil J Ethnopharmacol 2011 134 725 732 10.1016/j.jep.2011.01.027 21277967

[B18] Mensor LL Menezes FS Leitão GG Reis AS Dos Santos TC Coube CS Leitão SG Screening of Brazilian plant extracts for antioxidant activity by the use of DPPH free radical method Phytother Res 2001 15 127 130 10.1002/ptr.687 11268111

[B19] Hili P Evans CS Veness RG Antimicrobial action of essential oils: the effect of dimethylsulphoxide on the activity of cinnamon oil Lett App Microbiol 1997 24 269 275 10.1111/j.1574-6941.1997.tb00444.x 9134774

[B20] Clinical and Laboratory Standards Institute (CLSi) Methods for Dilution Antimicrobial Susceptibility Tests, fourth ed 2008 Wayne, PA, USA Approved Standard, M7-A4

[B21] Mahboubi M Bidgoli FG Antistaphylococcal activity of *Zataria multiflora* essential oil and its synergy with vancomycin Phytomedicine 2010 17 548 550 10.1016/j.phymed.2009.11.004 20171067

[B22] Ocvirk R Murphy P Franklin KB Abbott FV Antinociceptive profile of ring A-reduced progesterone metabolites in the formalin test Pain 2008 138 402 409 10.1016/j.pain.2008.01.019 18343034

[B23] Shibata M Ohkubo T Takahashi H Inoki R Modified formalin test: characteristic biphasic pain response Pain 1989 38 347 352 10.1016/0304-3959(89)90222-4 2478947

[B24] Shin S Jeon JH Park D Jang JY Joo SS Hwang BY Choe SY Kim Y Anti-inflammatory effects of an ethanol extract of *Angelica gigas* in a carrageenan-air pouch inflammation model Exp Animals 2009 58 431 436 10.1538/expanim.58.431 19654443

[B25] Mehta D Malik AB Signaling Mechanisms Regulating Endothelial Permeability Physiol Rev 2006 86 279 367 10.1152/physrev.00012.2005 16371600

[B26] Fraga CG Oteiza PI Dietary flavonoids: Role of (-)-epicatechin and related procyanidins in cell signaling Free Rad Biol Med 2011 51 813 823 10.1016/j.freeradbiomed.2011.06.002 21699974

[B27] García-Lafuente A Guillamón E Villares A Rostagno MA Martínez JA Flavonoids as anti-inflammatory agents: implications in cancer and cardiovascular disease Inflam Res 2009 58 537 552 10.1007/s00011-009-0037-3 19381780

[B28] Wittchen ES Endothelial signaling in paracellular and transcellular leukocyte transmigration Front Biosc 2009 14 2522 2545 10.2741/3395 PMC2654604 19273217

[B29] Kusuda M Inada K Ogawa TO Yoshida T Shiota S Tsuchiya T Hatano T Polyphenolic constituent structures of *Zanthoxylum piperitum* fruit and the antibacterial effects of its polymeric procyanidin on methicillin-resistant *Staphylococcus aureus* Biosc Biotechnol Biochem 2006 70 1423 1431 10.1271/bbb.50669 16794323

[B30] Clinical and Laboratory Standards Institute (CLSi) Performance standards for antimicrobial susceptibility testing, 18th informational supplement 2008 Wayne, PA, USA: CLSI document M100-S18

[B31] Webster D Taschereau P Belland RJ Sand C Rennie RP Antifungal activity of medicinal plant extracts; preliminary screening studies J Ethnopharmacol 2008 115 140 146 10.1016/j.jep.2007.09.014 17996411

[B32] Cos P Vlietinck AJ Berghe DV Maes L Anti-infective potential of natural products: how to develop a stronger in vitro ‘proof-of-concept’ J Ethnopharmacol 2006 106 290 302 10.1016/j.jep.2006.04.003 16698208

[B33] Van Bambeke F Mingeot-Leclercq MP Struelens MJ Tulkens PM The bacterial envelope as a target for novel anti-MRSA antibiotics Trends in PharmacolSc 2008 29 124 134 10.1016/j.tips.2007.12.004 18262289

[B34] Zampini IC Cuello S Alberto MR Ordoñez RM D’ Almeida R Solorzano E Isla MI Antimicrobial activity of selected plant species from “the Argentine Puna” against sensitive and multi-resistant bacteria J Ethnopharmacol 2009 24 499 505 19467313 10.1016/j.jep.2009.05.011

